# Centromere Localization for Bighead Carp (*Aristichthys nobilis*) through Half-Tetrad Analysis in Diploid Gynogenetic Families

**DOI:** 10.1371/journal.pone.0082950

**Published:** 2013-12-20

**Authors:** Chuankun Zhu, Yanhong Sun, Xiaomu Yu, Jingou Tong

**Affiliations:** 1 State Key Laboratory of Freshwater Ecology and Biotechnology, Institute of Hydrobiology, Chinese Academy of Sciences, Wuhan, China; 2 University of Chinese Academy of Sciences, Beijing, China; Auburn University, United States of America

## Abstract

Gene-centromere (G-C) mapping provides insights into structural and behavioural properties of chromosomes. In this study, G-C mapping using microsatellite markers and meiogynogenetic (meiotic gynogenetic) families were performed in bighead carp (*Aristichthys nobilis,* 2N = 48), which belongs to Cyprinidae. A total of 218 microsatellites were selected across 24 linkage groups (LGs) of a recently well-defined genetic linkage map for bighead carp, with 151 being heterozygous in at least one of six dams in diploid meiogynogenetic families. After tests for Mendelian segregation in two diploid control families, 103 microsatellites were used for G-C distance calculation in 383 gynogens. The second division segregation frequency (*y*) was computed through half-tetrad analyses, and the values ranged from 0 to 0.97 (mean 0.40). High G-C recombination frequencies (over 0.667) were observed in 18 (17.5%) of the loci examined, which revealed a low level of chiasma interferences compared with other fishes studied previously. Distribution of G-C distances across LGs ranged from 0 cM to 48.5 cM (mean 20 cM) under the assumption of complete interference. All 24 centromeres were localized according to their closest-related microsatellites at 95% confident intervals. The average distance between centromeres and their closest-linked markers was 6.1 cM with 15 out of 24 LGs having a distance below 5 cM. Based on the centromere positions in this study, we proposed a formula of 24 m/sm+24 t/st chromosomes with 92 arms for bighead carp, which was mostly in accordance with a previously reported karyotype for bighead carp (24 m/sm+24 st). These results of centromere localization provide a basic framework and important resources for genetics and comparative genomics studies in bighead carp and its closely-related cyprinid species.

## Introduction

Genetic mapping provides a framework for studies of quantitative trait loci (QTL) identification [1], comparative genome mapping [2], genome assembly and position-based cloning [3]. Genetic maps have been constructed for many aquatic animals over the past decade [4], [5], [6]. Nevertheless, genetic linkage maps only provide a reference to landmarks along the physical surface of a chromosome without a knowledge of centromere position [7]. Gene-centromere (G-C) mapping has become an essential tool to resolve the structural and behavioral property of chromosomes not only for its ability to localize centromeres, but also for its potential to define fixed points within linkage groups of DNA markers, identify proximal and distal marker genes, distinguish both chromosomal arms and investigate the interference phenomenon [8]. Comparisons of G-C maps among species can give insights into mechanisms of chromosome rearrangements occurred during speciation, which is useful information for evolution studies [9].

The approach of half-tetrad analysis is the basis for G-C mapping, only if two of the four chromatids from a single meiosis were recovered, half-tetrad analysis could be performed [10]. Meiotic gynogenesis (Meiogynogenesis) provides a way to recover these two chromatids. By inhibiting release of the second polar body in fish and some other aquatic organisms, gynogenetic diploids or triploids can be produced and applied for the analysis of meiosis II (MII) half-tetrads [11]. With the nondisjunctions of the second polar body during MII, a dam heterozygous at a particular co-dominant marker locus should produce only two homozygous gametes when no crossovers occur between the marker and the centromere, but if crossovers occurred during meiosis I (MI), heterozygous gametes should emerge. The recombination rate between marker and centromere can be readily estimated by measuring the proportion of heterozygous gynogens which is also a measure of the frequency of the second division segregation (*y*) [12]–[13]. For the G-C distance estimation, three mapping functions including complete interference [14], 50% interference [15] and no interference [16] can be applied.

Most G-C mapping studies in aquatic animals were based on allozyme markers around a decade ago [14], [17]–[18]. With the advantage and popularity of co-dominant DNA markers, microsatellite-centromere (M-C) mapping has recently been reported in many aquatic animals, including rainbow trout *Oncorhynchus mykiss* [19], zebrafish *Denio rerio* [20], loach *Misgurnus anguillicaudatus* [21], Japanese eel *Anguilla japonica* [22], large yellow croaker *Pseudosciaena crocea* [23], turbot *Scophthalmus maximus* [24], half-smooth tongue sole *Cynoglossus semilaevis* [25], walking catfish *Clarias macrocephalus* [26] and so on. However, most previous studies estimated G-C distances and only localized a few centromeres due to the lack of enough co-dominant markers on the genetic map and/or insufficient half-tetrad analyses. Centromeres were located on all linkage groups (LGs) in only a few aquatic animals including zebrafish [12], rainbow trout [27] and Pacific abalone *Haliotis discus hannai* [28].

Bighead carp (*Aristichthys nobilis*) is one of the most important aquaculture fish in China and has been introduced into many other countries for plankton control and human consumption [29]. However, population resources of bighead carp have sharply declined during the past decades which highlighted the urgent need of genetic improvement for bighead carp, therefore, a well-defined genetic linkage map and centromere map in this species are highly desirable. Actually, we estimated the M-C distances for 66 microsatellites in bighead carp previously [30], however, only one centromere was positioned onto LG4 of the bighead carp genetic map constructed by Liao et al. (2007) with low resolution and limited number of microsatellites [31]. Recently we generated a second-generation genetic linkage map for bighead carp (2N = 48) with 659 microsatellites assigned onto 24 LGs [32]. In this study, we aimed to localize centromeres onto all 24 LGs of our new genetic map for bighead carp. Meanwhile, we intended to analyze chiasma interferences and recombination rates in bighead carp chromosomes. The information obtained from G-C mapping and centromere localization would be useful for understanding the genome structure and chromosome evolution of the species.

## Materials and Methods

### Ethics Statement

Usage of bighead carp was permitted by the Zhangdu Lake Fish Farm Managing Committee. All the experimental animal programs applied in this study were approved by the Institute of Hydrobiology, Chinese Academy of Sciences’ Animal Care and Use Committee (IHBACUC), and followed the experimental basic principles. A slight fin tissue from the parents and control families was sheared under MS222 anesthesia, progenies of the experimental families were sacrificed with anhydrous ethanol, and all efforts were made to minimize suffering.

### Experimental Families and Genomic DNA Extraction

Parental females and males of bighead carp were selected from broodstocks of the Zhangdu Lake Fish Farm (Wuhan, China) to generate experimental families. Totally, six gynogenetic families (A–F) and two normal diploid control families (G, H) were produced by artificial propagation during 2008–2011. Gynogenetic families were obtained through a previous method [33] with slight modifications. Briefly, bighead carp eggs were fertilized with UV-irradiated common carp (*Cyprinus carpio*) sperm which was four times diluted by Hank’s solution, and then immersed into 4°C water bath immediately to inhibit the release of the second polar body. For the bighead carp control families, eggs from a dam were fertilized with sperm from a sire to produce normal diploid progenies. Fertilized eggs were hatched in circulating water with a temperature of approximately 25°C. Gynogens were raised in laboratory tanks and fed with hatched *Artemia* cysts until sampling, while control families (G, H) were raised in muddy ponds. At the age of one month after hatching, fingerlings of each meiogynogenetic family were sampled and preserved in anhydrous ethanol at 4°C. Fin tissues were sampled for control families G and H at the ages of 3 years and 1 year old, respectively. Fin clips from each parental fish were also sampled. Genomic DNA was extracted from alcohol-preserved fin tissues and fingerlings following a standard phenol-chloroform protocol [34].

### Microsatellite Selection and Genotyping

A set of microsatellite markers were chosen from each of the 24 LGs of a recently well-defined genetic linkage map for bighead carp [32] to position centromeres. A total of 218 microsatellites were initially selected across all LGs for potential uses in this study. Polymorphism of these markers were tested in the dams of six gynogenetic families, and those polymorphic markers were then amplified in control families to verify their Mendelian expectations (1∶1, 1∶2:1 and 1∶1:1∶1) which was statistically confirmed by chi-square (*χ^2^*) test (*p*<0.05). Microsatellites in accordance with the Mendelian segregations were applied to perform analyses of M-C distances and centromere positioning. Some of these microsatellite markers had trans-species ability to amplify common carp-specific alleles, therefore, they were used to verify the success rates of meiogynogenesis for six experimental families of bighead carp.

The microsatellite markers were amplified through PCR in a total volume of 12.5 µL, containing 1.25 µL of 10×reaction buffer, 0.4 µL of dNTP (2.5 mmol/L), 1 U of *Taq* polymerase (TaKaRa, Japan), 0.4 µL of forward and reverse primer mixture (2.5 µmol/L), 20–50 ng of template DNA and 9.4 µL of sterile water. A 96 well thermal cycler (Veriti, ABI) was used to perform PCR amplifications using the following program: 94°C denaturing for 5 min, followed by 35 cycles of 94°C for 35 s, optimal annealing temperature (Table S1) for 35 s and 72°C for 40 s, and a final extension at 72°C for 8 min. PCR amplicons were separated through 10% polyacrylamide gel electrophoresis and visualized by JS-A380 gel imaging system (PeiQing, China) after stained by ethidium bromide (EB).

### G-C Distance Calculation

As we found no any gynogens that had heterozygous genotypes in all microsatellites used in this study, therefore, parental tetratypes would not play a role in the calculation of G-C recombination rate (frequency of second meiotic division segregation, *y*). Because the *y* value was defined as the proportion of heterozygous recombinant genotypes in meiotic gynogens for each locus [12], [14], then calculation of the *y* in this study could be expressed with this formula: y = *Ne*/(*Ne*+*No*), where *Ne* is the number of heterozygotes (AB, parental ditypes) and *No* is the number of the two homozygotes (AA and BB, nonparental ditypes) in a mapping family. If a marker was informative in two or more families and showed unbiased *y* values among these families, an average was taken as the *y* value for this marker. Differences of G-C recombination frequencies among families were tested by contingency *χ^2^* test (*p*<0.05). Homozygosity induced by one generation of gynogenesis, which is defined as fixation index (*F*), was calculated by *F* = 1−*y* [35].

G-C distances (*x*) were calculated in three different mapping methods: i) complete interference, where *x* = 100(*y*/2), assuming that one recombination exchange precludes additional crossovers [14]; ii) 50% interference, assuming a reduction of interference with *x* = [ln (1+*y*)−ln (1−*y*)]×100/4 [15]; iii) zero interference, based on the equation *x* = −[ln (1−*y*)]×100/2, assuming no chiasma interference [16].

The correlation of distances between markers in this study and corresponding distances in the linkage map [32] was analyzed through the Spearman Rank Correlation Test in the software SAS 9.1.3 (SAS Institute Inc., Cary, NC, USA). In addition, regression analysis between marker distances estimated in the two maps was performed using the Microsoft Excel.

### Centromere Localization in Bighead Carp Map

A consensus genetic linkage map for bighead carp [32] was used as a framework to localize centromeres. Two markers at both ends of each LG, as close to extremes as possible, were initially selected to ascertain centromere orientation along the chromosomal axis. If large G-C distances were detected from both terminal markers, a centromere was considered to be localized at an internal position between the two markers. Then a few more markers, as closer as possible to the potential centromere region, were further selected for a more precise localization. After establishment of the centromere orientation along the chromosome axis, consistency between the recombination frequency and the position in linkage map of bighead carp was analyzed for each marker as described in a previous study [24]. Those markers, whose positions in the linkage map were apparently incongruent with diploid gynogenetic segregation, were not applied for centromere localization.

The relative position of each marker to the centromere was estimated by considering the minimum number of multiple recombination events, under the hypothesis of complete interference. The 95% confidence interval for a probable centromere region was estimated according to the formula *y*/N ±1.96{[(*y*/N)(1−*y*/N)]/N}^1/2^, where *y* is the number of heterozygous progenies for the indicated locus, and N is twice the number of progenies [12]. If a locus amplifies no heterozygous genotypes in the samples of half-tetrad individuals, *y* in the second term of the above formula is set equal to 1 [12].

As examples, the patterns and frequencies of crossovers, and the values of chiasma interference in chromosomes were estimated in selected LGs of the bighead carp genetic map, following the methods described previously [7], [28].

## Results

### Mendelian Segregation

Of the 218 markers, 151 were heterozygous in at least one of the six dams of gynogenetic families, and these markers were amplified in alternative control families to verify their segregation patterns. To obtain more reliable segregation data in meiogynogenetic families, those markers with possible null alleles were eliminated for recombination analysis, no matter they were in accordance with Mendelian expectations or not. Of the genotypic ratios for 151 markers, 103 were in accordance with Mendelian expectations at 5% level after sequential Bonferroni correction for multiple tests (Table S1), and the rest 48 markers (31.8%) segregated distortedly. These 103 markers were then individually genotyped in at least one of the six meiogynogenetic families, and their genotypic data in control and gynogenetic families were shown in Table S2 and Table S3, respectively.

### Verification of Meiogynogenesis

Common carp-specific alleles were observed in a total of 13 progenies from families A, B, E and F (Table 1), while no such alleles were detected in families C and D. Therefore, the success rates of meiogynogenesis ranged from 87.3% in family B to 100% in families C and D (Table 1), with an average success of 96.6% in this study. Those hybrid progenies were eliminated from recombination analysis.

**Table 1:** Verification for six meiogynogenetic families of bighead carp used for microsatellite-centromere mapping in this study.

Family	A	B	C	D	E	F	Average
No. of available markers	39	15	47	42	21	37	34
No. of discerning markers^a^	22	15	20	25	7	35	21
No. of progenies	72	55	50	60	80	80	66
No. of hybrids	2	7	0	0	1	3	2
Success rates ofgynogenesis (%)	97.2	87.3	100	100	98.8	96.3	96.6

a Microsatellite markers with unique alleles for males of common carp.

### Microsatellite–centromere Recombination

The number of heterozygous microsatellites segregated in gynogenetic families A to F were 39, 15, 47, 42, 21 and 37 respectively, and G-C distances were estimated initially based on these loci (Table 1). The ratio between two homozygote genotypes was compared with expected 1∶1 for each locus in corresponding gynogenetic family, most cases of which met the expectation (Table S3). However, 19 cases showed segregation distortion at the 5% level (Table S3). Because unequal proportion of homozygotes could affect the estimation of G-C distances, these 19 cases were excluded from the calculations of G-C distances. In addition, none of the markers which were genotyped in at least two of the six gynogenetic families showed biased values of G-C recombination frequencies after *χ^2^* test (*p*<0.05) (data not shown).

The overall heterozygote frequencies ranged from 0 (*Arsd298* in LG10, *HysdE11798-1* in LG4, *Hysd942-1* in LG20, *Arsd542* in LG15 and *Arsd276* in LG12) to 0.97 (*HysdE4406-1* in LG12) with an average of 0.40, corresponding to a fixation index (*F*) of 0.60 after one generation of gynogenesis. Low G-C recombination frequencies (*y*<0.1) were detected in 18 markers (17.5%, distributing in 14 LGs), whereas 18 markers (17.5%, distributing in 16 LGs) showed high recombination frequencies over 0.667, a value expected for independent segregation between a given locus and its centromere under the assumption of zero interference. These results indicated the existence of interference after a single chiasma formation in some chromosomes of bighead carp.

**Figure 1:**
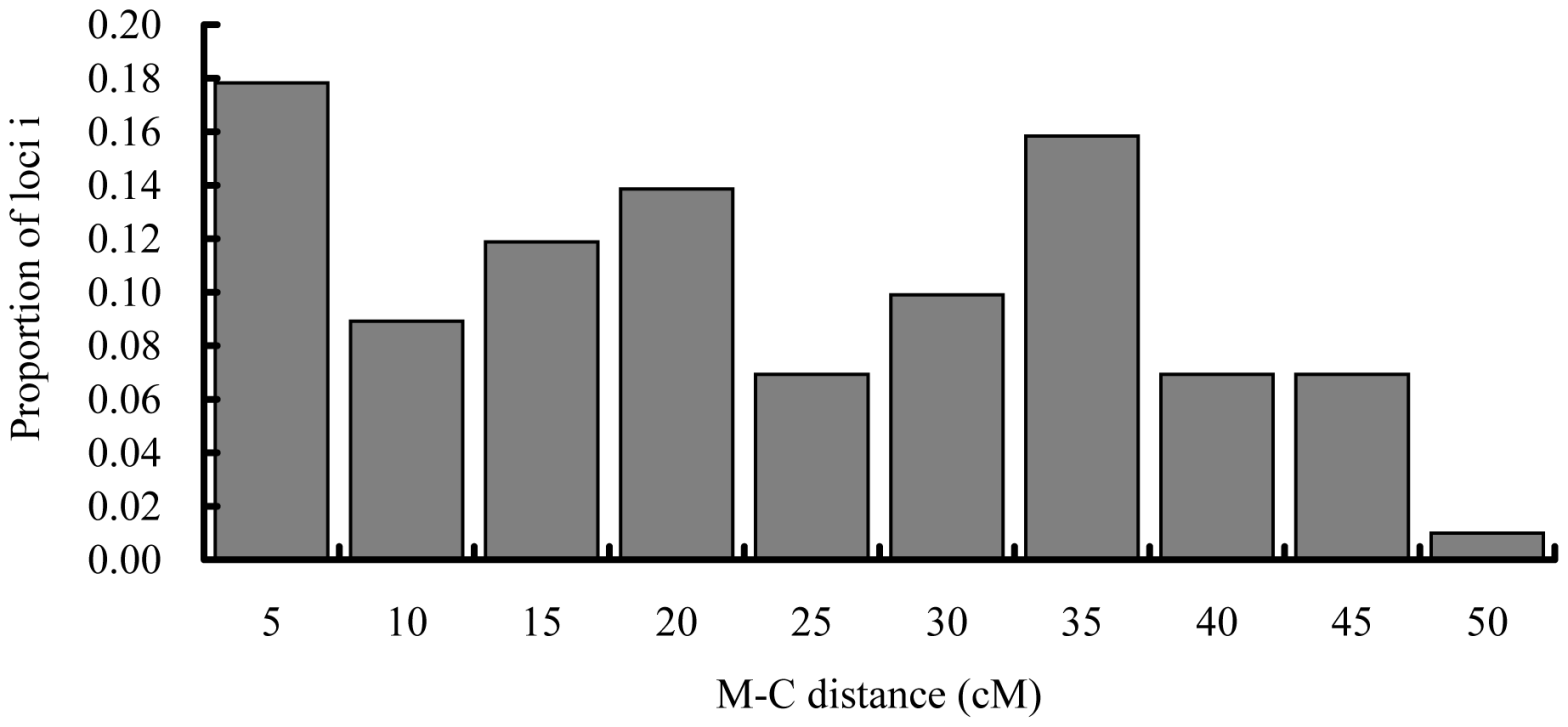
Distribution of microsatellite-centromere distances for 103 loci segregating in gynogenetic diploid bighead carp under the assumption of complete interference.

The estimated G-C distances under the assumptions of complete interference (*y*/2), 50% interference (Kosambi function) and zero interference (Haldane function) ranged from 0 to 48.5 cM, from 0 to 104.6 cM and from 0 to 175.3 cM, respectively (Table S3). When the *y* value was low, G-C distances were very similar under three conditions, however, with the increase of *y* values the differences in map distances also raised among three assumptions (Table S3). The frequency histogram of G-C distances under the complete interference showed that, of all microsatellites assessed, 17.5% located in the centromeric region, 17.5% in the telomeric region and 65% in the intermediate region of the bighead carp chromosomes (Fig. 1).

Correlation analysis of marker distances between the G-C map and linkage map revealed that genetic distances between markers were significantly related between the two maps under the 1% level (rho = 0.389, p = 0.000). Regression between G-C distance and linkage distance, forced through the origin, had a slope of 0.771; but if those distances longer than 30 cM were excluded, then calculated slope was 0.931, which was much closer to 1 (Fig. S1). Of those 6 marker pairs with linkage distances above 30 cM, 5 pairs emerged below the 1∶1 regression line (Fig. S1).

### Centromere Localization

**Figure 2:**
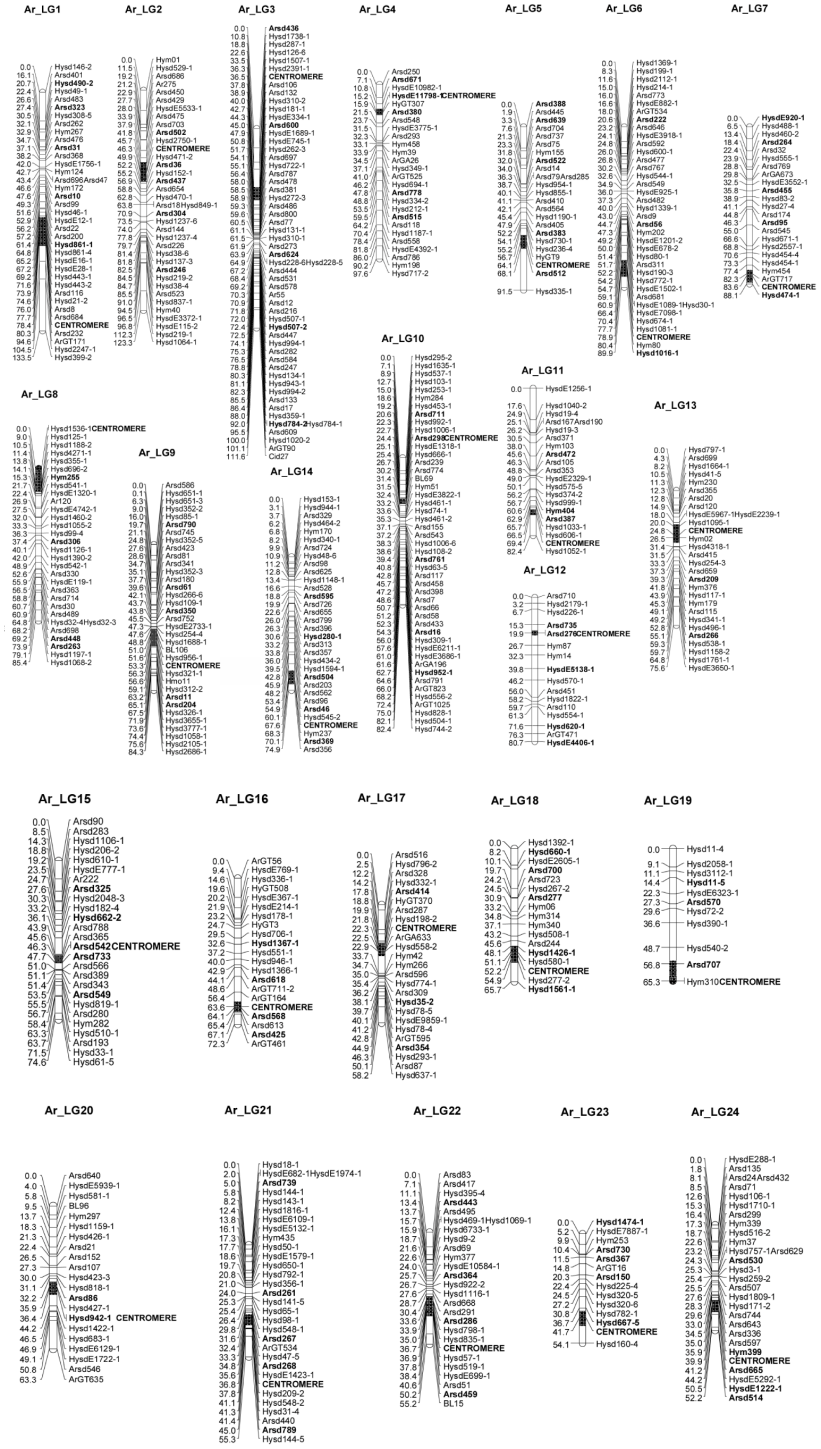
Centromere positioning for 24-C mapping. Microsatellites used in this study are in bold characters and centromere positions estimated by half-tetrad analyses are shown as black rectangles (95% confidence intervals).

Comparisons of marker positions in LGs of the linkage map [32] with the observed recombination frequencies of diploid gynogenetic segregation data indicated incongruity at 10 markers distributing in 9 LGs (Table S3), and these markers were not used for centromere positioning. Based on G-C distances estimated under the complete interference assumption, centromeres were successfully positioned onto all 24 LGs of the second generation genetic linkage map for bighead carp (Fig. 2). The position of a given centromere was shown as the region indicated by 95% confidence intervals which were inferred from the marker near the centromere. Based on the positional information of centromeres, 24 LGs [32] could be divided into two types, with the metacentric/submetacentric (m/sm) in one half and the telocentric/subtelocentric (t/st) in another half. Therefore, a formula of 24 m/sm +24 t/st chromosomes were identified as the karyotype for bighead carp in this study (Table 2).

**Table 2:** Classification for 24[32] based on the centromere positions obtained in this study.

	Centromere position (cM)	LG length (cM)	LG type^a^
LG1	78.4	133.5	m/sm
LG2	46.3	123.3	m/sm
LG3	36.5	111.6	m/sm
LG4	15.2	97.6	t/st
LG5	64.1	91.5	m/sm
LG6	78.9	89.9	t/st
LG7	83.6	88.1	t/st
LG8	0	85.4	t/st
LG9	56.3	84.3	m/sm
LG10	24.4	82.4	m/sm
LG11	69.4	82.4	t/st
LG12	19.9	80.7	t/st
LG13	24.8	75.6	m/sm
LG14	67.6	74.9	t/st
LG15	48.3	74.6	m/sm
LG16	63.6	72.3	t/st
LG17	22.3	58.2	m/sm
LG18	52.2	65.7	t/st
LG19	65.3	65.3	t/st
LG20	23.2	63.3	m/sm
LG21	36.8	55.3	m/sm
LG22	36.7	55.2	m/sm
LG23	41.7	54.1	t/st
LG24	39.9	52.2	t/st

a m/sm: metacentric/submetacentric; t/st: telocentric/subtelocentric.

### Crossover and Interference

**Figure 3:**
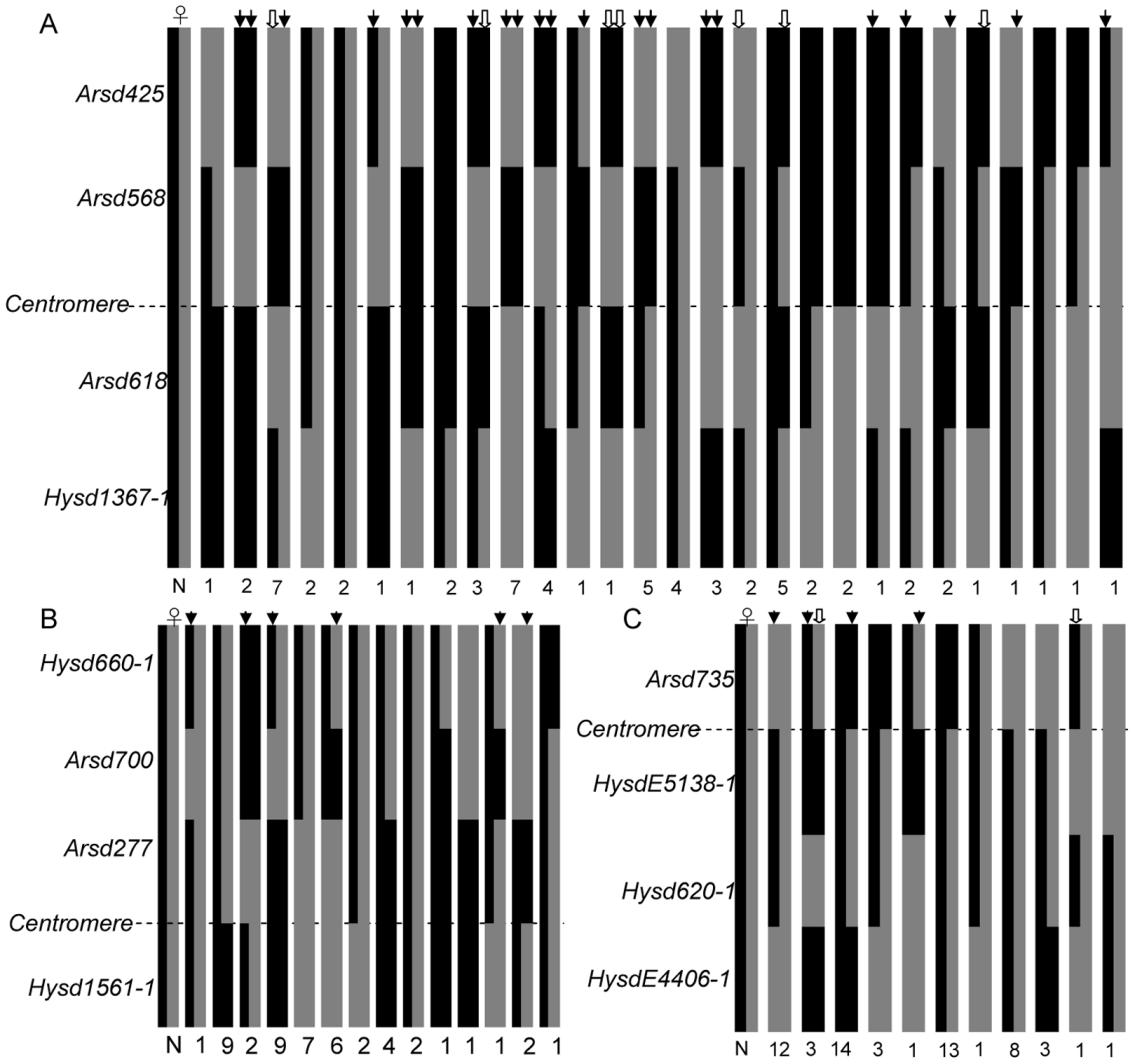
The crossover patterns in chromosomes corresponding to LG16 in family A (A), LG18 in family C (B) and LG12 in family D (C) of bighead carp. The horizontal dotted lines stand for centromeres in the three LGs. The bars on the left of each figure represent two chromosomes in dams of the three families. Each chromosome segment in gynogens of each family is filled with grey or black color to indicate the genotype at the distal marker; changes in color within a bar represent exchanges between non-sister maternal chromatids. Double crossovers and triple crossovers are labelled by arrows and hollow arrows, respectively. N is the frequency of each four-locus genotype in three gynogenetic families.

We examined half-tetrad genotypes in details for three cases (individuals with missing data were not included), i.e. LG16 in family A, LG18 in family C and LG12 in family D, to evaluate the distribution patterns of crossovers between markers (Fig. 3). In LG16, among the 134 chromosomes from 67 gynogens of family A, 20 were non-crossover chromosomes (NCO; 14.9%), 31 were single crossover (SCO; 23.1%), 63 were double crossover (DCO; 47.0%) and 20 were triple crossover (TCO; 14.9%). In LG18 (family C, 96 chromosomes), 29 NCO (30.2%), 46 SCO (47.9%), 21 DCO (21.9%) and zero TCO were detected. In LG12 (family D, 120 chromosomes) the recombination patterns were as follows: 49 NCO (40.8%), 37 SCO (30.8%), 30 DCO (25%) and 4 TCO (3.3%). Although frequencies and distribution patterns of crossovers are different in these 3 LGs, TCO has always the lowest frequency.

The interference value between two markers based on the DCO was estimated in LG18 as an example. The proximal marker *Arsd700* and distal marker *Hysd660-1* in this LG theoretically produced nine genotypic combinations in family C (Table 3). Two DCO chromosomes were estimated from one gynogen, being heterozygous at *Arsd700* but homozygous at *Hysd660-1*. The expected number of DCO chromosomes in the absence of interference at each interval is (25/48)×(1–42/48)×48 = 3.125. Thus, the coefficient of coincidence is 2/3.125 = 0.64, corresponding to an interference value of 0.36.

**Table 3:** Recombination between *Hysd660-1* and *Arsd700* in the family C of gynogenetic diploid bighead carp.

	*Arsd700*			
*Hysd660-1*	AA	AB	BB	total
AA	3	0	0	3
AB	10	24	8	42
BB	0	1	2	3
Total	13	25	10	48

## Discussion

### Segregation Distortion

Segregation distortions have been observed in many aquatic organisms, especially in marine fish and shellfish [24], [36]–[37]. In this study, the proportion of distorted markers was unexpectedly high (31.7%), almost twice of the distortion ratio (16%) observed in the construction of the second generation genetic linkage map for bighead carp [32]. In sea cucumber (*Apostichopus japonicus*) only 2.3% microsatellites deviated from the Mendelian ratios in the M-C study using 24-h larvae [13], but the ratio rose to 23.8% in the construction of genetic linkage map using two-year-old *A. japonicus* [38]. Similar phenomenon has also been noted in flat oyster *Ostrea edulis* [39], Pacific oyster *Crassostrea gigas* [40] and Pacific abalone [28]. All of these studies demonstrated that segregation distortion was minimal at the early zygote stage and increased during development.

Significant differences in the proportions of segregation distortions in bighead carp were most probably caused by stages of sampling. The ages for progenies of the control families in this study was 3 years old for family G and 1 year old for family H, respectively, while the progenies for preparation of the genetic linkage map [32] were only one month old. Fish individuals with low viability may be alive during larval stage, but would vanish gradually along with the expression of lethal or deleterious genes, which would cause segregation distortion [9]. Another reason for high ratio of segregation distortion in this study may be that those markers with potential null alleles were also treated as distorted markers.

We found that the ratio between two non-recombinant homozygous genotypes significantly deviated from the expected Mendelian ratio of 1∶1 in 19 cases involving 18 of the 103 loci and 5 mapping families. Surprisingly, families A and F occupied 15 of these 19 cases with 10 in family A and 5 in family F, respectively. As suggested by previous studies, one of the two homozygous genotypes may link to recessive lethal or deleterious genes causing a significant segregation distortion in diploid meiogynogenetic families [9], [24]. The high distortion proportion in family A and F indicated that their dams may have experienced inbreeding, since they were more sensitive to gene homozygosity.

### The Fixation Index

The fixation index (*F*), calculated by 1-*y,* is an evaluation for the extent of homozygosity [9]. The value of *F* in this study (0.60) was similar to that estimated in our previous study for bighead carp (0.523) [30], but much higher than Japanese eel (0.355) [22], walking catfish (0.357) [26], large yellow croaker (0.414) [23] and turbot (0.425) [24]. The *F* obtained in this study is 2.4 times of the inbreeding coefficient after one generation of sib-mating (*F* = 0.25), indicating that meiogynogenesis could provide an effective means for rapid inbreeding in bighead carp.

### G-C Distances and Chiasma Interference

The second division frequencies (*y*) in bighead carp gynogenesis ranged from 0 to 0.97 with an average of 0.40, which is similar to that (0.477) in our previous study [30], but lower than many other fishes [22]–[24], [26]. This suggested that the rates of crossovers between homologous chromosomes may be markedly lower in bighead carp than those in majority of fishes. A proportion of 17.5% loci showed *y* above 0.667, which is lower than ratios detected previously in bighead carp (25.76%) [30], large yellow croaker (45.5%) [23], turbot (50%) [24] and walking catfish (60%) [26]. These results strongly suggested that the rate of chiasma interference in bighead carp is significantly lower than above fish species.

G-C distances in this study ranged from 0 to 48.5 cM under the assumption of complete interference, suggesting that the microsatellites are widely distributed from proximal (centromeric) to distal (telomeric) regions of bighead carp chromosomes. The distribution pattern of G-C distances in bighead carp was similar to that of Pacific abalone [28], which was also obtained based on a well-defined genetic linkage map. Markers from a higher resolution genetic map would cover wider region of chromosomes, which allowed the results of M-C mapping to be more accurate. This viewpoint could be supported by a wider distribution region of G-C distances (0–48.5 cM) in this study than that in a previous one (2.85–43.75 cM) [30] in the bighead carp.

Significant correlation of G-C distances and genetic linkage map distances indicated that high interference of crossovers may exist in bighead carp genome, as suggested by previous studies that this correlation is a reflection of complete or nearly complete interference of crossovers in the recombination [41]. This positive correlation was also observed in other aquatic animals such as Pacific oyster [42] and salmon [43]. Regression slope for markers separated by 30 cM or less in this study was 0.931, similar to that previously reported in Pacific oyster (1.06) [42], suggesting that G-C distances between markers are accurate. Therefore, selected markers with small distances on genetic linkage map ensure reliable results of the G-C mapping in this study.

Chiasma interference is common in fish, which may be due to mechanical difficulties of double cross-over in fish with relatively small size of chromosomes [14]. In this study, through the crossover analyses in LG12, LG16 and LG18 we speculated that both frequencies and distribution patterns of crossovers vary among LGs and crossovers with three or more times were only in a small proportion of the recombination categories in bighead carp. Similar phenomena were also observed in Pacific abalone [28]. The interference rate estimated from joint segregation for *Arsd700* and *Hysd660-1* in LG18 was 0.36, a medium value when compared with Pacific abalone (0.18) [28] and rainbow trout (0.78) [14]. Because interference and inter-regional genetic distance on chromosomes is inversely correlated [44], interference values would be higher between markers with a shorter distance, and lower between markers with a longer distance.

### Localization of Centromeres

The identification of centromere positions is a perfection for genetic linkage maps, and is also an initial step towards understanding the composition and structure of the centromeric region as well as the whole genome. Mainly due to the lack of well-defined genetic linkage maps using co-dominant markers, centromeres have been located only in very limited aquatic species so far. Zebrafish is the first fish in which all 25 centromeres were localized on genetic linkage maps [12], followed by rainbow trout [27], Atlantic halibut *Hippoglossus hippoglossus* [45] and turbot [24]. Only one centromere was located onto the first generation bighead carp genetic map [31] in our previous study [30]. In sea cucumber, centromeres on two LGs, LG3 and LG20, were localized in the genetic linkage map [13], and all 18 centromeres were positioned in Pacific abalone [28].

The closest distance between marker and centromere is very important in the estimation of centromere regions, the closer of the distance the more accurate of the centromere regions [12]. The closest distances between microsatellites and centromeres in this study ranged from 0 to 17.0 cM with an average of 6.1 cM. In addition, 62.5% LGs (15 out of 24) had closest-linked markers to centromeres (≤5 cM), which was higher than that in turbot (30.8%, 8 out of 26 LGs) [24]. Since 6.1 cM is near the criterion for the closest linkage to centromeres (≤5 cM) [24] and majority of bighead carp LGs contained closeset-linked markers, therefore, positioning of centromeres in this study is credible.

Based on the results of our M-C mapping, 24 bighead carp LGs can be divided into two types, with a proposed karyotype formula of 24 m/sm+24 t/st chromosomes for diploid genome. This is in coincidence with a previous formula of 24 m/sm+24 st proposed by Almeida-Toledo et al. (1995) [46], but different from other karyotypes reported for bighead carp [47]–[50]. In fact, results of karyotype studies in bighead carp were different from each other. Many factors may affect karyotypic data: firstly, small size of fish chromosomes may bring high deviation in length measuring; secondly, various measuring precisions may alter results; and finally, techniques of metaphase preparation and chromosome spread may also affect final karyotypic results [46].

Four of the five previous karyotypes had 96 chromosome arms for diploid bighead carp (48 for haploid) [46]–[49]. However, the number of chromosome arms in this study was 92 (46 for haploid), since two LGs (LG8 and LG19) were telocentric. The absence of chromosome arms may be due to the lack of linked and segregated microsatellite markers on these two short arms of the bighead carp LGs. If a high-density genetic map were available for bighead carp and more markers could be selected from LG8 and LG19 for M-C mapping in future, all feasible chromosome arms would be detectable through half-tetrad analyses.

Since genes (microsatellites) can be mapped in relation to their centromeres, G-C mapping allows us to compare gene orders between bighead carp and other fishes, which can provide insight into the mechanism of chromosomal rearrangements [9], and will be also helpful for further studies on chromosomal and genomic evolutions in bighead carp and other cyprinids. With the help of G-C mapping and centromere localization, genes closely-linked with centromeres can be mapped into linkage maps [12], which would provide references for gene positional cloning, integration between genetic and physical maps, and quantitative trait locus identification in bighead carp.

## Conclusions

G-C recombination frequencies of 0–0.97 (mean 0.40) were obtained for bighead carp based on 103 microsatellites through half-tetrad analysis. The patterns and proportions of chiasma interferences were different among LGs, and the rates of both recombination and chiasma interference in bighead carp were lower than those reported in other fishes. Under the assumption of complete interference, all 24 centromeres were localized onto their respective LGs of our second generation genetic map for bighead carp with 95% confident intervals. Based on centromere positions in this study, we proposed a karyotypic formula of 24 m/sm+24 t/st for bighead carp chromosomes. The results of this M-C mapping study successfully integrated the centromere map and genetic linkage map in bighead carp, which provide valuable information for consolidation of genetic map and physical map in future. This study would be also helpful for studies on genome structure, chromosome evolution, and positional cloning for genes of interest in this aquaculture species.

## Supporting Information

Figure S1 **Regression of inter-marker distances between G-C map in this study and the genetic linkage map of bighead carp** [**32**] **, for all 77 marker-pairs formed by 103 microsatellites.** The solid line is regression line for all marker-pairs and the dotted one is for those with genetic distances shorter than 30 cM on the genetic linkage map. Slopes of the two lines are marked.
(TIF)

Table S1 **Summary information for microsatellite markers used in this study.**
(XLS)

Table S2 **Genotypic distribution of 103 microsatellites in two control families (G and H) of bighead carp.**
(XLS)

Table S3 **Microsatellite–centromere (M-C) recombination rates (second meiosis segregation frequency) and M-C map distances based on 103 microsatellites segregating in six meiogynogenetic families of bighead carp.**
(XLS)
