# Supplementary figures and images for: Centromere Localization for Bighead Carp (Aristichthys nobilis) through Half-Tetrad Analysis in Diploid Gynogenetic Families
Source: PLoS One. 2013 Dec 20;8(12):e82950. doi: 10.1371/journal.pone.0082950 (PMC3869750; doi:10.1371/journal.pone.0082950)

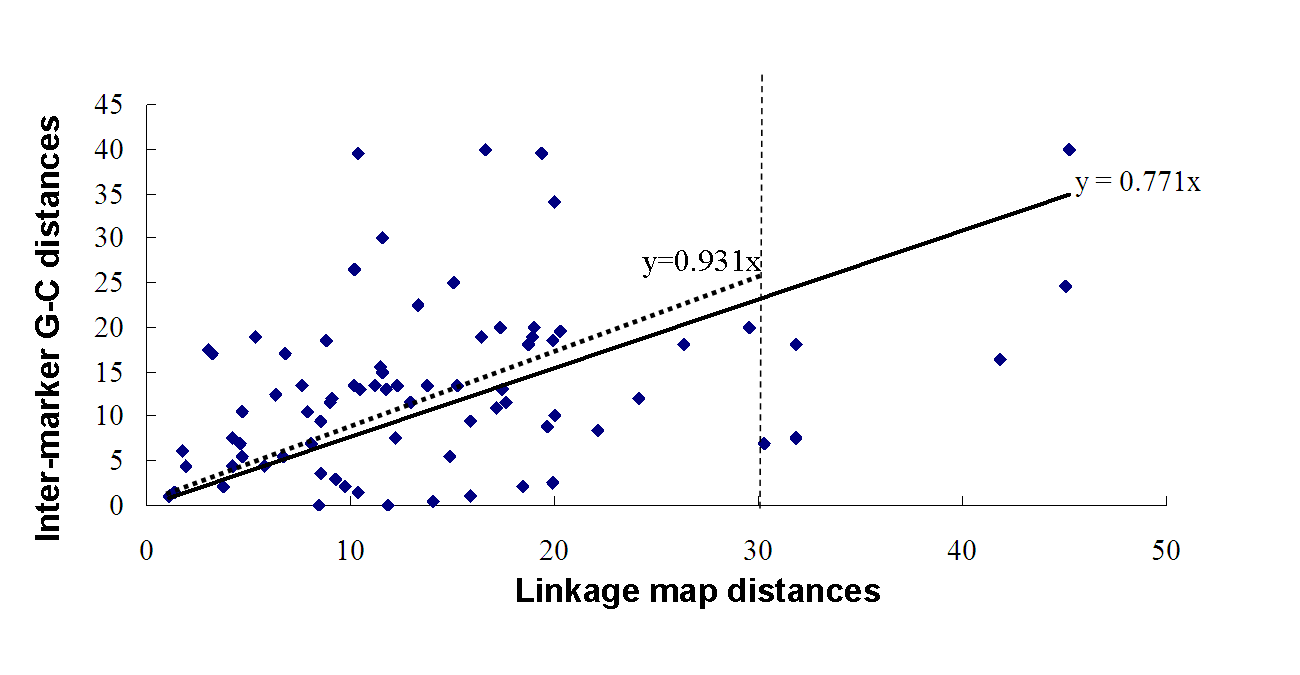

Supplement: Figure S1 — Regression of inter-marker distances between G-C map in this study and the genetic linkage map of bighead carp [32] , for all 77 marker-pairs formed by 103 microsatellites. The solid line is regression line for all marker-pairs and the dotted one is for those with genetic distances shorter than 30 cM on the genetic linkage map. Slopes of the two lines are marked. (TIF) [file pone.0082950.s001.tif]
